# Estrogenic Effects of Several BPA Analogs in the Developing Zebrafish Brain

**DOI:** 10.3389/fnins.2016.00112

**Published:** 2016-03-24

**Authors:** Joel Cano-Nicolau, Colette Vaillant, Elisabeth Pellegrini, Thierry D. Charlier, Olivier Kah, Pascal Coumailleau

**Affiliations:** Research Institute in Health, Environment and Occupation, Institut National de la Santé et de la Recherche Médicale U1085, SFR Biosite, Université de Rennes 1Rennes, France

**Keywords:** 17β-estradiol, aromatase, cyp19a1b, bisphenol, BPA, hypothalamus, endocrine disruption

## Abstract

Important set of studies have demonstrated the endocrine disrupting activity of Bisphenol A (BPA). The present work aimed at defining estrogenic-like activity of several BPA structural analogs, including BPS, BPF, BPAF, and BPAP, on 4- or 7-day post-fertilization (dpf) zebrafish larva as an *in vivo* model. We measured the induction level of the estrogen-sensitive marker *cyp19a1b* gene (Aromatase B), expressed in the brain, using three different *in situ/in vivo* strategies: (1) Quantification of *cyp19a1b* transcripts using RT-qPCR in wild type 7-dpf larva brains exposed to bisphenols; (2) Detection and distribution of *cyp19a1b* transcripts using *in situ* hybridization on 7-dpf brain sections (hypothalamus); and (3) Quantification of the *cyp19a1b* promoter activity in live *cyp19a1b*-GFP transgenic zebrafish (EASZY assay) at 4-dpf larval stage. These three different experimental approaches demonstrated that BPS, BPF, or BPAF exposure, similarly to BPA, significantly activates the expression of the estrogenic marker in the brain of developing zebrafish. *In vitro* experiments using both reporter gene assay in a glial cell context and competitive ligand binding assays strongly suggested that up-regulation of *cyp19a1b* is largely mediated by the zebrafish estrogen nuclear receptor alpha (zfERα). Importantly, and in contrast to other tested bisphenol A analogs, the bisphenol AP (BPAP) did not show estrogenic activity in our model.

## Introduction

Estrogens play important roles in many developmental and physiological processes (Boon et al., 2010) and references therein, including for brain development (Hojo et al., 2008; Hill and Boon, 2009; Bondesson et al., 2014; Coumailleau et al., 2015). Bisphenol A (BPA) is a well-known chemical compound that mimic and interfere with the actions of endogenous estrogens and thus act as an endocrine disruptor (Krishnan et al., 1993; Gould et al., 1998; Paris et al., 2002; Kitamura et al., 2005; Richter et al., 2007; Wetherill et al., 2007; Vogel, 2009; Grignard et al., 2012). BPA is a chemical used in many industrial and commercial applications including in the production of polycarbonate plastics and epoxy resins, present in a variety of consumer products such as food-packaging materials, toys, thermal and recycled papers, compact discs, impact-resistant safety equipment, and medical devices to name a few (Vinggaard et al., 2000; Vandenberg et al., 2010; Liao and Kannan, 2011; Staples et al., 2011; Huang et al., 2012; Liao et al., 2012a,c). BPA rapidly became one of the most produced and used chemicals worldwide (about 3.4 million tons per year). The widespread use of BPA has resulted in its detection in environment (Huang et al., 2012; Liao et al., 2012b), in food (Schecter et al., 2010; Liao and Kannan, 2013), and in human biological sample (Sun et al., 2004; Calafat et al., 2005, 2009; Vandenberg et al., 2010; Zalko et al., 2011; Fenichel et al., 2012; Liao et al., 2012a; Vandenberg et al., 2012). Epidemiological studies, along with laboratory studies in many species including primates, provide increasing support that environmental BPA exposure can be harmful to humans and is associated with a wide range of effects in humans, rodents, and wildlife. Indeed, BPA exposure is linked to numerous adverse health concerns including development, diabetes, obesity, cardiovascular, reproductive disorders, behavioral troubles, chronic respiratory and kidney diseases, and carcinogenesis, likely linked to the endocrine disrupting effects (Vandenberg et al., 2012; Rochester, 2013; Rezg et al., 2014). Owing those potential health concerns, Canada (2009), USA (2010), and the European Union (2011) prohibited the use of BPA in the manufacture of polycarbonate feeding bootles for infants. In France, since January 2015, BPA is forbidden in any food or beverage packaging.

Such restrictions on BPA usage recently led manufactories to use alternative bisphenols. Such alternatives include among others bisphenol AF, bisphenol F, and bisphenol S (Liu et al., 2012). For instance, BPS is found in canned soft drinks, canned foods (Vinas et al., 2010; Gallart-Ayala et al., 2011) and in thermal receipt papers (Becerra and Odermatt, 2012; Liao et al., 2012c). BPAF is also incorporated into the production of fluoropolymers, fluoroelastomers, and in a variety of polymers that are used in the manufactoring of electronic devices and plastic optical fibers (Yang et al., 2012). Recent large-scale quantitative studies have identified, in addition to BPA, increasing concentrations of various bisphenols such as BPAF, BPAP, BPF, BPS, BPB, BPZ, and BPP in food products in the United States (Liao and Kannan, 2013). In addition, BPB, BPF, and BPS were also detected in indoor dust in the USA and in several Asian countries (Liao et al., 2012b). However, despite the increasing use of BPA analogs, there is limited information on potential toxicity and endocrine-disrupting activities of these molecules. However, few BPA analogs present some endocrine disrupting activity, as assessed by *in vitro* analysis. For instance, it was shown that BPS and BPAF can bind to estrogen receptors and subsequently exert estrogenic activity at the transcriptional level using cell culture and binding assays (Hashimoto et al., 2001; Kitamura et al., 2005; Kuruto-Niwa et al., 2005; Matsushima et al., 2010; Grignard et al., 2012). Although the estrogenic potential of few BPA analogs have been demonstrated *in vitro*, the *in vivo* potential endocrine-disrupting activity of these compounds remains largely unknown. Recent physiological studies suggest that at least a few BPA analogs have the potential to interfere and disrupt the normal functions of endocrine system in various organisms (Feng et al., 2012; Ji et al., 2013; Naderi et al., 2014; Yang et al., 2014; Eladak et al., 2015). A growing number of studies have shown that BPA has a negative impact on neural development and on the onset of neurological disorders, likely associated to its endocrine-disrupting activities (reviewed in Kajta and Wojtowicz, 2013; Leon-Olea et al., 2014; Negri-Cesi, 2015). To our knowledge, very limited work has assessed estrogenic activity of BPA analogs during brain development, and/or in adult brain. A recent study suggests that exposure to BPS might cause hyperactivity and brain changes in developing zebrafish (Kinch et al., 2015).

In the present study, we assessed the potential *in vivo* estrogenic activities of various BPA analogs and their effects on the central nervous system using the developing zebrafish brain. The developmental pattern of the zebrafish is particularly well-studied (Briggs, 2002) and the species is a widely used model to evaluate the potential adverse effects of chemicals present in the environment and to define the mechanisms underlying the endocrine-disrupting activities (Segner, 2009). Indeed, numerous estrogen-sensitive proteins have been identified in zebrafish, including the liver-produced yolk proteins Vitellogenin 1 and 3 (encoded by vtg1 and vtg3 genes), and the brain-specific aromatase B (AroB), encoded by the brain specific *cyp19a1b* gene, and change in their expression can be used as biomarker for estrogen or xenoestrogen exposure (Kausch et al., 2008; Ruggeri et al., 2008; Levi et al., 2009; Chung et al., 2011; Lam et al., 2011; Hao et al., 2013). We and others have shown that the *cyp19a1b* gene is specifically expressed in a very specific brain population, the radial glial cells, that serves as progenitors during embryonic and adult neurogenesis (for review see Diotel et al., 2010; Coumailleau et al., 2015; Pellegrini et al., 2015). In addition, the presence of functional estrogen response elements in *cyp19a1b* proximal promoter region allows for a strong transcriptional upregulation by estrogens (E2) and xenoestrogens such as ethinyl estradiol (EE2) and BPA (Le Page et al., 2006; Sawyer et al., 2006; Chung et al., 2011; Brion et al., 2012). Thus, the *cyp19a1b* gene can be used *in vivo* as a biomarker of xenoestrogen effects on the central nervous system in developing and adult zebrafish.

In the present work, we investigated the effects of various BPA analogs on *cyp19a1b* expression in developing zebrafish brain exposed from 0 to 1 day post-fertilization (0–1 dpf) to 4–7 dpf. We used 3 different *in situ/in vivo* approaches: (1) quantitative RT-PCR to monitor the expression levels of *cyp19a1b* in wild type larvae (7 dpf); (2) *cyp19a1b in situ* hybridization to precisely analyse the induction and distribution of *cyp19a1b* transcripts in wild type 7-dpf larvae, and (3) the quantification of the brain fluorescence of *cyp19a1b*-GFP transgenic 4-dpf larvae as an *in vivo* assay (EASZY assay). We demonstrate that the majority of the tested bisphenol A analogs (BPS, BPF, and BPAF) induces *in vivo* significant expression of *cyp19a1b* in the brain of zebrafish at early developmental stages.

## Materials and methods

### Chemicals

Bisphenol analogs, including bisphenol A [BPA; 2,2-bis(4-hydroxyphenyl)propane; 99%), bisphenol F [BPF; 4,4′-dihydroxydiphenyl methane; 98%), bisphenol AF [BPAF; 2,2-bis(4-hydroxylphenyl)hexafluoropropane; 98%), bisphenol S [BPS; bis-(4-hydroxyphenyl)sulfone; 98%), bisphenol AP [BPAP; 4,4′-(1- phenylethylidene)bisphenol; 98%], were purchased from Sigma-Aldrich (St. Louis, MO) and TCI America (Portland, OR). E2 [17β-estradiol] and EE2 [17α-ethinylestradiol] were purchased from Sigma Aldrich (St. Louis, MO, USA). ICI 182 780 was purchased from Tocris Bioscience. Stock solutions were prepared in dimethyl sulfoxide (DMSO; Sigma) and kept at −20°C. Dilution series were freshly prepared before each experiment. The maximum volume of the solvent did not exceed 0.1% (v/v).

### Zebrafish maintenance and embryo/larva exposure

Animals were treated in agreement with the European Union regulations concerning the protection of experimental animals. This study was approved by the ethics committee (CREEA: Comité Rennais d'Ethique en matière d'Expérimentation Animale) under permit number EEA B-35-040. Zebrafish embryos were raised in our facilities (IFR 140, INRA SCRIBE, Rennes, France) in recirculated water kept at 28.5°C and spawned under standard conditions. Embryos were collected 2 h post-fertilization (hpf), and examined under a binoccular. Those embryos that had developed normally were selected and kept in several Petri dishes (zebra.s- c.edu/guides.html) in an incubator at 28.5°C (kept on a 14-h light, 10-h dark cycle).

For subsequent RT-qPCR and *in situ* hybridization analysis with wild type zebrafish, groups of 80 embryos (for each condition) were placed after 1 day post-fertilization (1-dpf) in a large glass flask containing 100 ml of embryonic medium (Mouriec et al., 2009b). Chemical treatments were performed by adding either DMSO alone (negative control), EE2 (positive control), or one of the tested bisphenols (BPA, BPS, BPF, BPAF, BPAP) diluted in water at indicated concentrations, thereby creating 7 experimental treatment conditions. The embryos were held in the exposure flasks until 7-dpf larva stage. For studies with the *cyp19a1b-GFP* transgenic zebrafish line (Tong et al., 2009; Brion et al., 2012), groups of 20 embryos (for each condition) were placed 2 h post-fertilization in crystallization dishes containing 25 ml medium and at indicated concentration of tested bisphenols analogs, EE2, or DMSO. Embryos were maintained in the exposure dishes until 4-dpf larva stage. During the treatment period, 100% of the exposure medium was renewed every 24 h (for both wild type and transgenic zebrafish). No mortality was observed for any treatments during the exposure period.

### Quantitative real-time PCR

After exposure, approximately 70 wild type heads were collected at 7 dpf (i.e., 6 days of exposure) for each experimental conditions (DMSO, EE2, BPA, BPS, BPF, BPAF, and BPAP) into 1.5 ml Eppendorf tubes, and frozen in liquid nitrogen. Tissue was sonicated (10 s, 3 times) in 250 μL Trizol Reagent (Invitrogen) and RNA extractions were carried out according to the manufacturer's protocol. Reverse transcription was carried out by incubating 2μg total RNA with 1μg of random primer oligonucleotides, 2.5ṁM dNTPs, and 50 U MMLV-RT (Promega) in the appropriate buffer for 10 min at 65°C and 60 min at 37°C. Quantitative Polymerase chain reaction (qPCR) experiments were performed in an iCycler thermocycler coupled to the MyiQ detector (Bio-Rad. Hercules, CA, USA) using iQ SYBR-Green Supermix (Bio-Rad) according to the manufacturer's protocol. The following primers were used: *ef1* (fw) 5′-AGCAGCAGCTGA GGAGTGAT-3′; *ef1* (rev) 5′-CCGCATTTGTAGATCAGATGG-3′; *cyp19a1b* (AroB; fw) 5′-TCGGCACGG CGT- GCAACTAC-3′; *cyp19a1b* (AroB; rev) 5′-CATACCTATGCATTGCAGACC-3′. For each condition, the RT-PCR experiment was run in triplicates. Expression levels of *ef1* mRNA were used to normalize *cyp19a1b* expression levels. Melting curve and PCR efficiency analyses were performed to confirm correct amplification. For quantification of PCR results, the threshold cycle (Ct) was determined for each reaction. Ct values for each gene of interest were normalized with the housekeeping gene *ef1*, using the ΔΔCt method. Normalized values were used to calculate the degree of induction or inhibition expressed as a “fold difference” compared to normalized control values.

### Brain sections and *In situ* hybridization

Larvae used for *in situ* hybridization experiments originated from the same exposition groups than RT-qPCR experiments. After exposure to the different conditions, 10 wild type 7-dpf larvae (for each treatment) were fixed overnight at 4°C in 4% paraformaldehyde, before embedding in parrafin. Serial thin transverse sections (8 μM) were placed on cryofrost slides and subjected to *in situ* hybridization experiments. Sense and antisense digoxigenin-labeled riboprobes for the *cyp19a1b* gene were transcribed using the Digoxigenin RNA labeling kit in accordance with the manufacturer's instructions (Roche, Mannheim, Germany) and as previously described (Menuet et al., 2005). The brain sections were processed for *in situ* hybridization using stringent conditions as previously published (D'Amico et al., 2011, 2013). After NBT/BCIP revelation, sections were counterstained with DAPI, and mounted in a drop of vectashield (Vector Laboratories). All sections were photographed with an Olympus PROVIS AX70 microscope with a digital camera (Olympus SP71), or a Nikon multizoom AZ100 macroscope with a DS-Ri1 color camera.

### *In vivo* imaging with the EASZY assay

Quantification of fluorescence in transgenic *cyp19a1b*-GFP zebrafish larva brain was performed according to Brion et al. (2012). In this assay, estrogenic activity is detected in living 4 days-old larvae (treated or not with an estrogen mimic compound) from the observation of the reporter gene fluorescence in the radial glial cells. After exposure conditions (see above), live tg (*cyp19a1b*-GFP), 4 dpf larvae (20 specimens per condition) were observed in dorsal view and each specimen was photographed using a Zeiss AxioImager.Z1 fluorescence microscope equipped with a AxioCam Mrm camera (Zeiss GmbH, Göttingen, Germany). All photographs were taken using the same parameters: only the head was photographed under a X10 objective, with a 134 ms exposure time and maximal intensity. Photographs were analyzed using the Axiovision Imaging software and fluorescence quantification was performed using the ImageJ software (http://rsbweb.nih.gov/ij/). For each picture, the integrated density was measured, i.e., the sum of the gray-values of all the pixels within the region of interest. A gray-value of 290 was defined as background value. For each micrograph the fluorescence fold induction of fluorescence was calculated comparing the integrated pixel density with the average fluorescence induction obtained in the control group.

### Plasmid constructions

The zfER-α, zfER-β1, and zfER-β2 expression vectors correspond to Topo-pcDNA3 expression vector (Invitrogen, San Diego, CA, USA), containing the coding regions of each zebrafish estrogen receptor cDNA as previously described (Menuet et al., 2002). The *cyp19a1b*-Luciferase plasmid consists of 500 bp of the proximal promoter region of zebrafish *cyp19a1b* gene, containing an ERE, coupled to the luciferase reporter gene (Menuet et al., 2002).

### Glial cell culture and transfection experiments

Human U251-MG glial cells were maintained in phenol red-free Dulbecco's Modified Eagle's Medium (DMEM; Life Technologies, Saint Aubin, France) supplemented with 10% fetal calf serum (Biowest, Nuaillé, France), 4 mM L-Glutamine (Gibco, Carlsbad, CA, USA) and 1 mM Na-Pyruvate (Life Technologies, Saint Aubin, France) and kept at 37°C and 5% CO_2_ atmosphere (Le Page et al., 2006). The medium was also supplemented with 20 U/mL penicillin, 20 μg/mL streptomycin, and 50 ng/mL amphotericin B (Gibco). For transfections, cells were plated in 24-well plates at a density of 25,000 cells/ml in the same medium, except the fetal calf serum was charcoal-treated and used at a concentration of 2.5%. Cells were transfected with 25 ng/well either the vector expression containing or not the the zfERα, zfERβ1, zfERβ2 coding region and cytomegalovirus [CMV]−β-Galactosidase, and 150 ng/well of the *cyp19a1b*-Luciferase reporter plasmid, using JetPEI as a transfection reagent (Polyplus Transfection, Illkirch, France). One day after transfection, U251-MG cells were treated with chemicals using same concentrations as *in vivo* experiments (10–9 M and 10–6 M, for EE2 and bisphenols, respectively) and DMSO as a vehicle (1/10.000). Luciferase activity was measured 24 h later (Luciferase assay system, Promega, Madison, WI, USA). β-Galactosidase activity was used to normalize transfection efficiency. Chemicals were tested in at least 3 independent experiments and each experiment was performed in triplicate.

### Zebrafish estrogen receptor competitive-binding assays

We also performed a competitive binding assay to test the binding properties of our compounds of interest with the three zfERs (Blair et al., 2000). The three zebrafish estrogen receptor proteins were synthesized using the Topo-pcDNA3 expression vector containing the coding region of zfERα, zfERβ1, and zfERβ2 (Menuet et al., 2002). The TNT Quick Coupled Transcription/Translation Systems kit (Promega, Madison, WI, USA) was used for synthesis of zfER proteins by adding 1 μg of each ER expression vector and according to the manufacturer's protocol. Efficiency of translation was assessed by SDS-PAGE (data not shown). After *in vitro* synthesis, 5 μl of zfERα, zfERβ1, or zfERβ2 were incubated overnight at 4°C with 10^−9^ M [^3^H]-E2 in absence or presence of increasing concentrations of radioinert E2 (10^−11^ M, 10^−10^ M, 10^−9^ M, 10^−8^ M, 10^−7^ M), BPA, BPF, BPS, BPAF, or BPAP (10^−10^ M, 10^−9^ M, 10^−8^ M, 10^−7^ M, 10^−6^ M, 10^−5^ M). The relative binding affinity for each compound was analyzed by their efficiency to move [^3^H]-E2 from the zfER binding site. Results were expressed as a percentage of displaced [^3^H]-E2 binding. The 10^−7^ M E2 containing 100-fold excess of radioinert E2 compared to [^3^H]-E2 was considered as the non-specific binding (Blair et al., 2000). IC50 were calculated using GraphPad Prism, version 6.07.

## Results

In this study, we tested the estrogenic potentials of various bisphenols (BPA, BPS, BPAF, BPF, and BPAP) using the estrogen sensitive biomarker *cyp19a1b* gene in zebrafish brain. Three different experimental approaches were used to validate our results: RT-qPCR on whole brain extract and *in situ* hybridization on brain sections in wild type zebrafish larvae (7-dpf), and the EASZY assay on *cyp19a1b*-GFP zebrafish larvae (4-dpf). In addition, we performed two *in vitro* assays including a transfection experiment in a glial cell context and a competitive binding assay, in order to identify the nuclear estrogen receptors mediating up-regulation of the *cyp19a1b* gene.

### Establishment of a proper BPA concentration inducing cyp19a1b gene in the brain of 7-dpf zebrafish larvae

**Figure 1:**
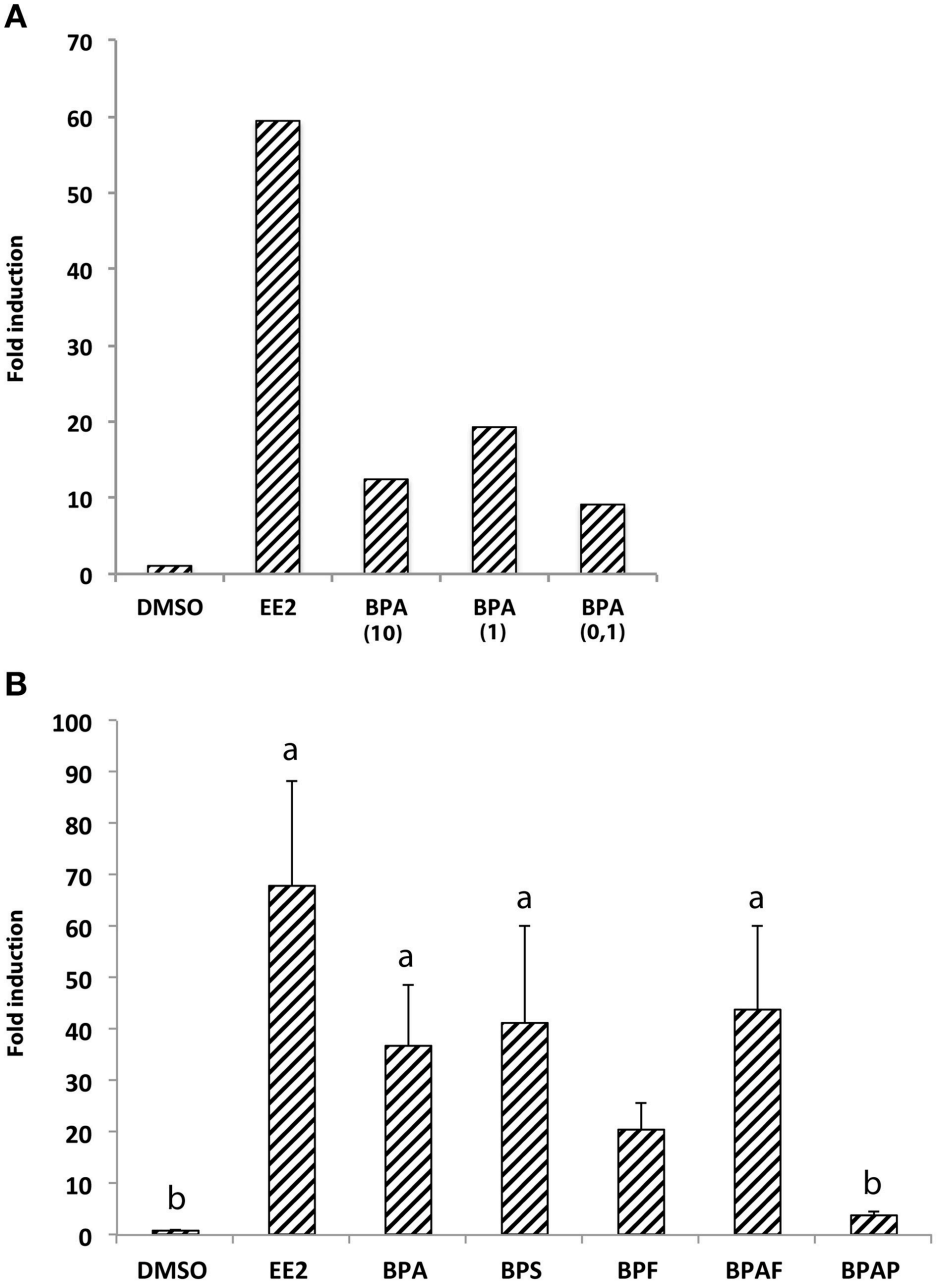
**(A)** Expression of *cyp19a1b* gene in 7-dpf larvae brains upon treatments with EE2 (1 nM) and three different concentrations of BPA (0.1, 1, or 10 μM). RNA levels were measured in triplicate by real-time quantitative RT-PCR of total RNA prepared from pooled animals (70 per condition). Fold induction was expressed relative to the solvent (DMSO); **(B)** Expression of *cyp19a1b* gene in 7-dpf larvae brains upon treatments with EE2, BPA, and four individual BPA analogs (BPS, BPF, BPAF, and BPAP). For each chemical the concentration used is 1 μM excepted for EE2 (1 nM) and the vehicle (DMSO). RNA levels were measured in triplicate by real-time quantitative RT-PCR of total RNA prepared from pooled animals (70 per condition). Fold induction was expressed relative to the solvent (DMSO); Data in **(B)** are presented as mean ± SEM of six separate egg exposures derived from six independent spawns. **a**: *p* < 0.05 vs. DMSO; **b**: *p* < 0.05 vs. EE2.

We first performed real-time RT-qPCR to determine the expression level of *cyp19a1b* mRNAs in the brain of 7-dpf zebrafish larvae exposed or not to bisphenol A (BPA) or ethinyl estradiol (EE2), a weak and a strong synthetic estrogenic compounds, respectively. Three different concentrations of BPA (0.1, 1, or 10 μM) were tested to identify the dose of BPA allowing the best *cyp19a1b* gene response, and compared to EE2 (1 nM) or control DMSO-treated larvae To overcome potential individual variations, we pooled 70 heads in each treatment group (DMSO, EE2, BPA 10, BPA 1, and BPA 0,1). In all treatment groups, no toxic or teratogenic effect was identified. Treated animals survived until 7-days larval stage and were identical to untreated larvae regarding morphology and motility (data not shown). After exposure, quantitative RT-PCR on brain extracts was then performed in triplicate as described in Materials and Methods. As shown in Figure 1A, exposure to EE2 induced a very strong overexpression (over 60-fold) of the *cyp19a1b* gene in the brain of 7-dpf larvae, in comparison to control larvae treated with DMSO only. A weaker induction, compared to EE2, was detected in larvae exposed to BPA ranging from 0.1 to 10 μM (Figure 1A). Interestingly, BPA concentration of 1 μM induced about 20-fold *cyp19a1b* transcripts levels, whereas 0,1, and 10 μM BPA concentrations had lower effects on *cyp19a1b* transcripts (9- and 12-fold inductions, respectively). As BPA was capable of stronger induction at a 1 μM concentration and did not caused notable toxicological effects, we therefore selected this specific concentration to analyze the effects of BPA analogs in subsequent experiments (RT-qPCR, *in situ* hybridization and EASZY assays). This concentration is also either below or similar to the bisphenols concentrations commonly used in studies on zebrafish embryos (Sun et al., 2009; Chung et al., 2011; Lam et al., 2011; Staples et al., 2011; Wu et al., 2011; Keiter et al., 2012; Ji et al., 2013; Saili et al., 2013; Tse et al., 2013; Wang et al., 2013; Naderi et al., 2014).

### Bisphenol A analogs induce cyp19a1b gene expression in wild type 7-dpf larva brain

The ability of BPA analogs to stimulate or not the expression of *cyp19a1b* estrogenic marker gene was then tested *in vivo* in the brain of wild type 7-dpf larvae. BPS, BPF, BPAF, and BPAP were tested at 1 μM on groups of 70 larvae. In addition, BPA (1 μM) and EE2 (1 nM) groups were used as positive controls, and a DMSO group as a negative control. Due to possible variations between egg batches, the entire experiment was repeated on six independent layings using the same standardized exposure protocols. Importantly, 1 μM treatments of BPA analogs did not affect the survival rate and the motility of exposed larvae (data not shown). In addition, no teratogenicity was observed all along the treatment and prior to the RT-qPCR analysis (data not shown). Figure 1B shows data obtained in the six independent experiments along with statistical analysis (Kruskal-Wallis). We found a general effect of the bisphenol analogs treatment on aromatase expression (*H* = 26.95, *p* < 0.0001). Dunn's *post-hoc* analysis showed a significant *cyp19a1b* induction by BPA, BPS, and BPAF in 7-dpf larvae (about 36-, 41-, and 43-fold induction compared to DMSO-treated larvae, respectively; Figure 1B). Although BPF exposure lead to an apparent increase in aromatase expression, this 20-fold up-regulation did not reach statistical significance. In contrast, BPAP had no effect on *cyp19a1b* gene.

### *In vivo* detection of cyp19a1b promoter activity using cyp19a1b-GFP transgenic larvae exposed to bisphenols

**Figure 2:**
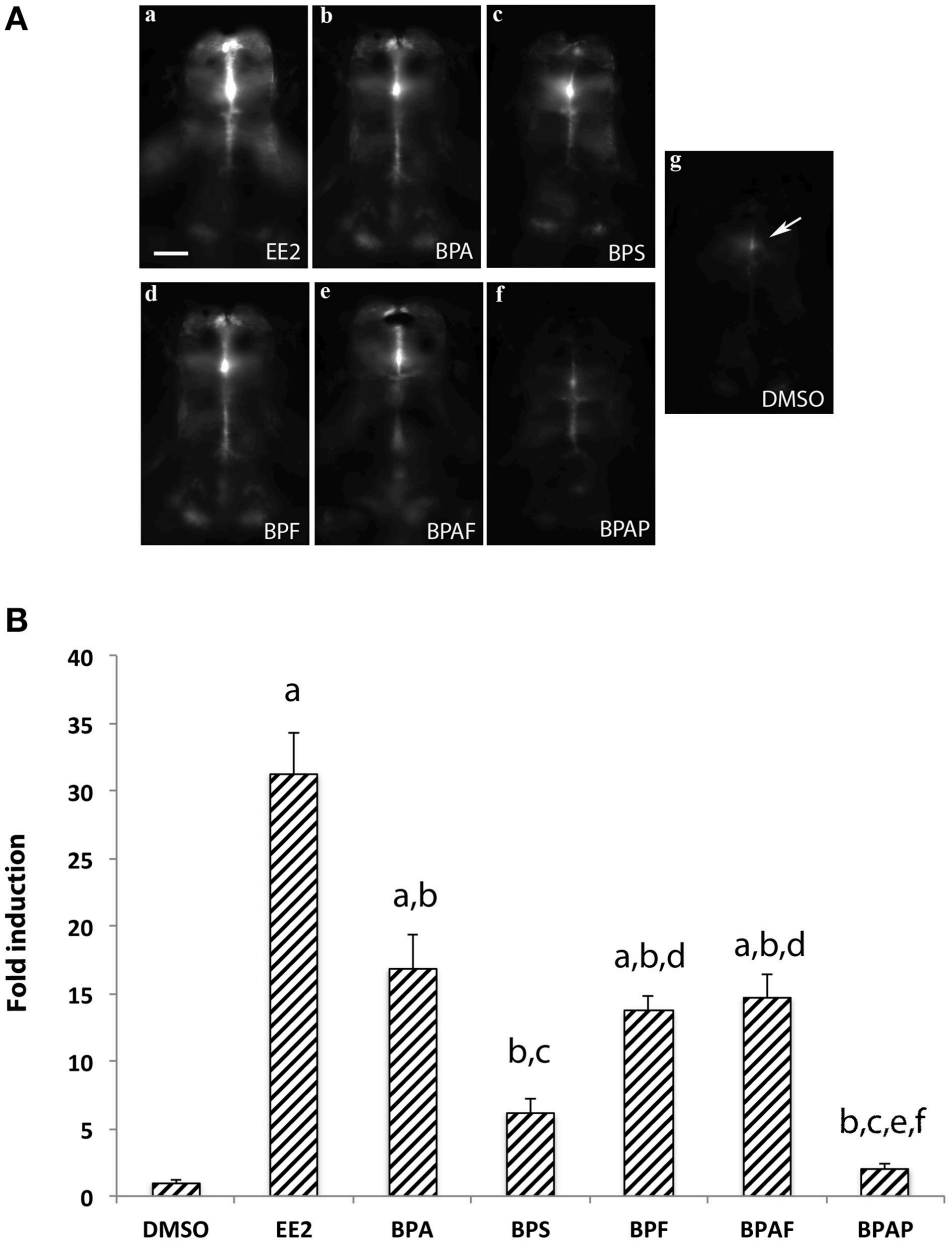
**(A)** *In vivo* imaging of 4-dpf old live transgenic *cyp19a1b*-GFP zebrafish larvae exposed to EE2, BPA, and various bisphenol A analogs.For each chemical the concentration used is 1 μM excepted for EE2 (1 nM) and the vehicle (DMSO). Dorsal views of the brains. Anterior is to the top. Bar = 20 μm. **(B)** Quantification of GFP fluorescence in zebrafish larvae exposed to the various compounds. Results are expressed as fold induction above control. Data re presented as mean ± SEM of 20 specimens per condition (*n* = 20). **a**: *p* < 0.05 vs. DMSO; **b**: *p* < 0.05 vs. EE2, **c**: *p* < 0.05 vs. BPA, **d**: *p* < 0.05 vs. BPS, **e**: *p* < 0.05 vs. BPF, **f**: *p* < 0.05 vs. AF.

To confirm the results obtained above, we tested the estrogenic activity of BPA analogs using the *cyp19a1b*-GFP transgenic zebrafish line, also named tg (cyp19a1b-GFP) (Tong et al., 2009). The use of tg (cyp19a1b-GFP) larvae was previously shown to be a very sensitive and fast assay (EASZY assay) to detect estrogenic activity (Brion et al., 2012; Petersen et al., 2013; Fetter et al., 2014). As described in Materials and Methods, experimental groups of 20 transgenic embryos were exposed for 4 days (from 2 h to 4 days post-fertilization) with 1 μM BPA, BPS, BPF, BPAF, or BPAP. In addition, control groups of transgenic embryos were exposed to either EE2 (1 nM) or DMSO alone. Figure 2A shows examples of the GFP signal generated in the whole brain by the different bisphenols together with the positive (EE2) and negative (DMSO) controls. Larvae treated only with DMSO (Figures 2Ag,B) show a basal GFP fluorescence, equivalent to those treated only with water (data not show). GFP induction occurs mostly in the midline of the brain, at the preoptic area level (arrow in Figure 2Ag). All other GFP fluorescence induction values were normalized in relation to this signal, considered as the basal activity of the *cyp19a1b* promoter (Figure 2B). Quantification of the signal and one-way ANOVA analysis revealed a significant overall effect of the treatment [*F*_(6, 85)_ = 35.43, *p* < 0.0001]. As expected, exposition of larvae to the synthetic estrogen EE2 (1 nM) strongly increased the GFP fluorescence intensity in the brain, attesting an important activity of the *cyp19a1b* promoter in the presence of EE2 (Figures 2Aa,B). An intense GFP signal is detected in the radial glial cells together with a much wider distribution from the telencephalon to the caudal hypothalamus (Figure 2A, compared Figure 2Aa and Figure 2Ag). As shown in Figure 2B, there was 31-fold induction of the GFP fluorescence in EE2-treated animals compared to DMSO-treated animals. The BPA also significantly increased the GFP fluorescence intensity (Figures 2Ab,B), albeit to a lower level compared to EE2 (16-fold induction compared to the DMSO control). Using this *in vivo* experiment, we also demonstrated that BPA analogs such as BPS, BPF, and BPAF also increased the GFP fluorescence in the brain (Figures 2Ac–e). Quantifications of the GFP signal reveals that fluorescence intensity for BPAF and BPF (14- and 13-fold inductions, respectively) was similar to BPA and significantly higher than the control (Figure 2B). The 6-fold induction by BPS did not reach statistical significance (Figure 2B). In contrast, larvae exposed to BPAP did not affect GFP fluorescence signal (Figures 2A,B). Taken together, these data show a correlation between the induction of the *cyp19a1b* promoter activity in the transgenic zebrafish line (trough measuring GFP fluorescence) and the *cyp19a1b* gene expression that we observed in RT-qPCR analysis in wild type larvae with BPA, BPS, BPF, and BPAF (Figure 1B). In addition, we also confirm that BPAP did not significantly induce *cyp19a1b* gene expression.

### Effects of bisphenols on cyp19a1b transcripts distribution in the developing zebrafish brain

**Figures 3, 4:**
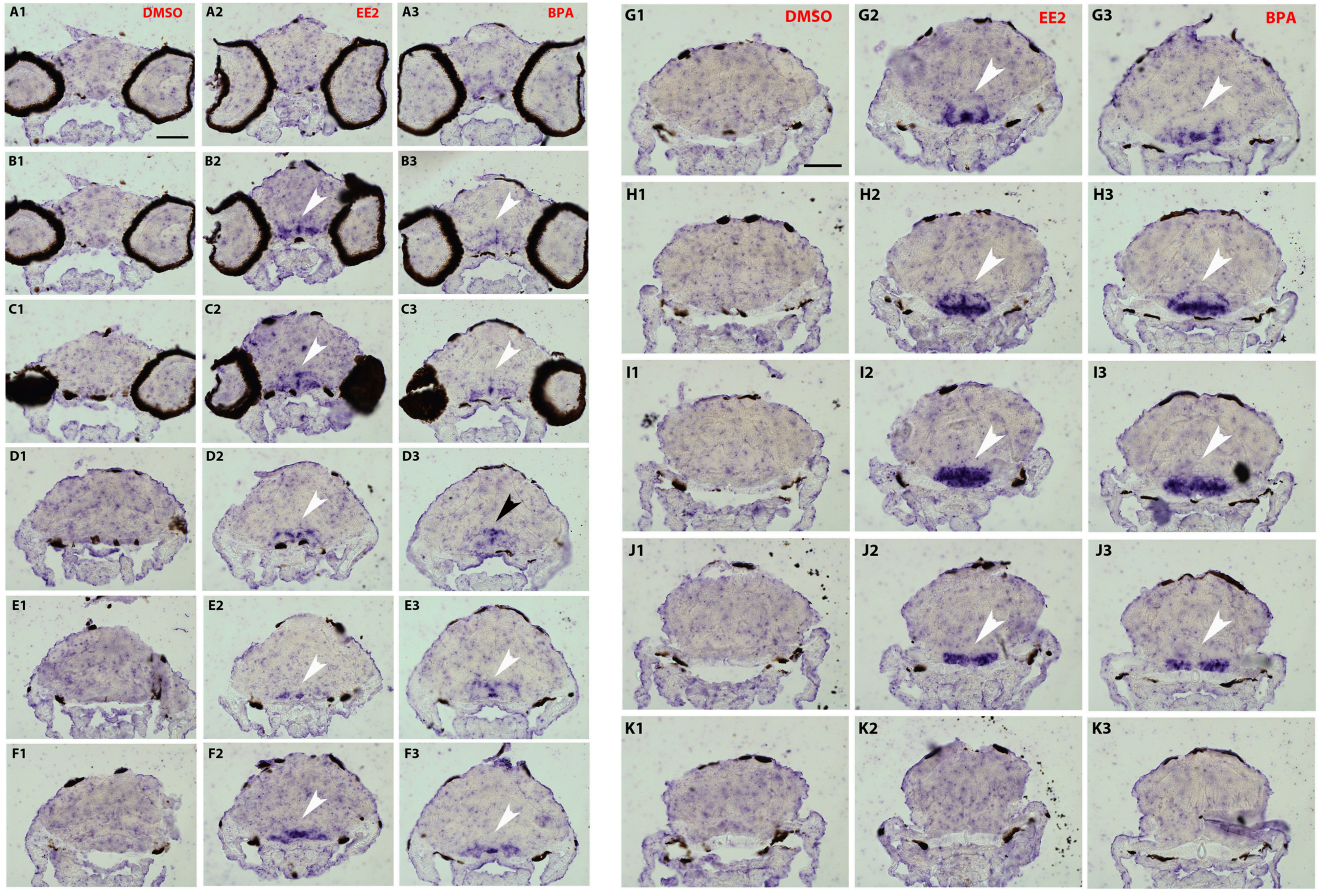
**Distribution of ***cyp19a1b*** transcripts in 7-dpf old zebrafish brains after treatments with EE2 (A2–K2) and BPA (A3–K3) and compared to the DMSO control (A1–K1)**. Images of transverse sections through the rostrocaudal axis of brains. Arrowheads highlight areas of labeling. For all images, dorsal is to the top. Scale bar = 50 μm.

To analyze a possible change in the distribution of *cyp19a1b* transcripts in the brain following bisphenol treatment, we performed *in situ* hybridization on serial and thin transverse sections of wild type exposed larva brains (7-dpf). We first compared *cyp19a1b* expression patterns in larvae exposed to EE2, BPA or DMSO alone. As shown in Figures 3, 4, *cyp19a1b* transcripts cannot be detected on thin sections along the rostral-caudal axis of brains that were only treated with DMSO (Figures 3, 4A1-K1). In contrast, EE2-treated larvae show a massive over-expression of the *cyp19a1b* transcripts in several and specific regions of the brain, notably in posterior telencephalon, preoptic area (arrows in Figures 3B2–D2) and caudal hypothalamus (lateral and posterior ventricular recesses; arrows in Figures 4G2–J2). Importantly, BPA-treated larvae also displayed strong presence of *cyp19a1b* transcripts in virtually identical regions than EE2-treated larvae (arrows in Figures 3, 4A3–K3). These *in situ* hybridization data confirm and extend previous studies showing that both estrogen and xenoestrogens increase *cyp19a1b* RNA levels in these brain regions (Menuet et al., 2005; Lassiter and Linney, 2007; Mouriec et al., 2009a; Tong et al., 2009; Chung et al., 2011).

**Figure 5:**
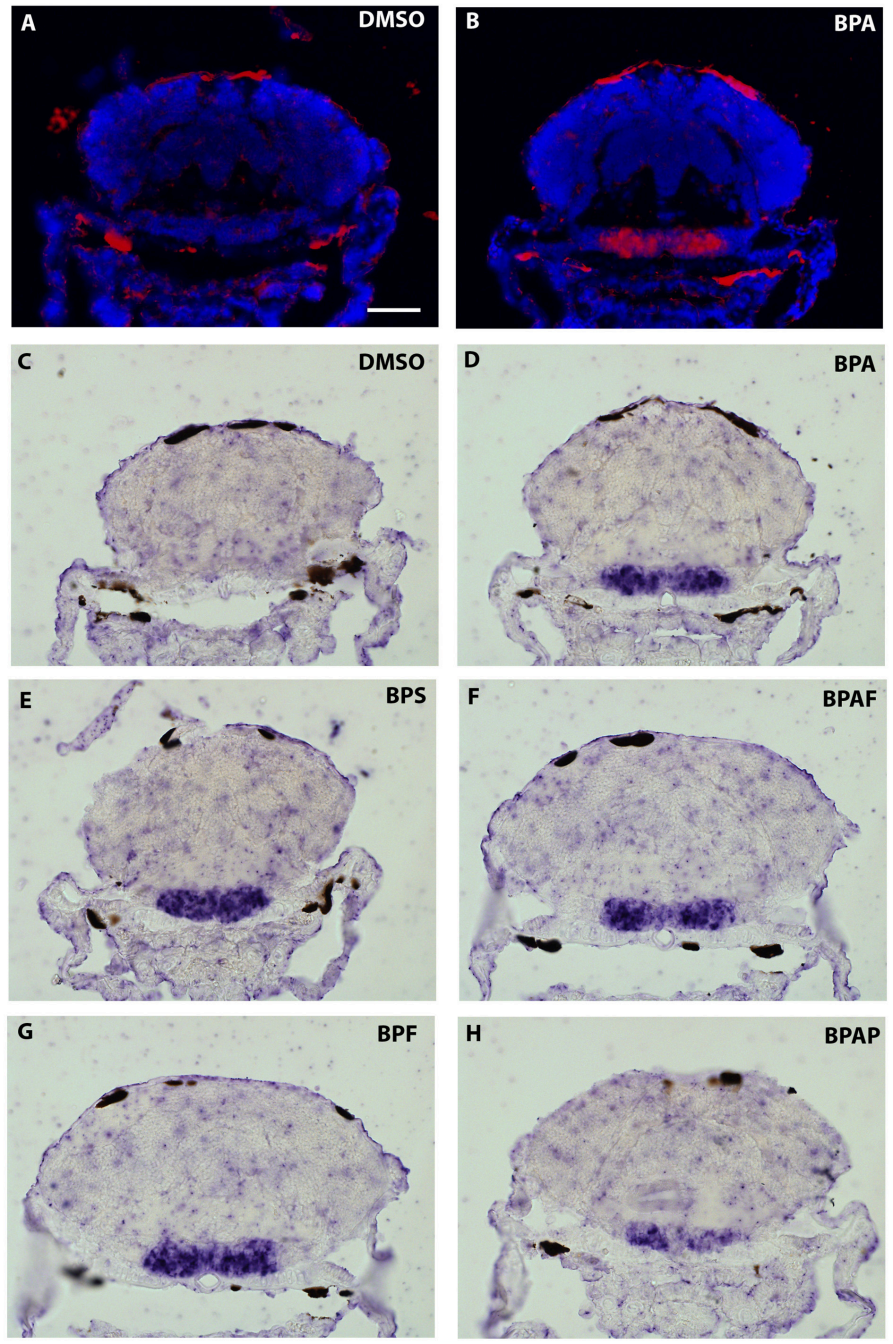
*****In situ*** expression of *cyp19a1b* at the level of the caudal hypothalamus (7-dpf old larvae) after treatments with various BPA analogs (E–H) and comparison with negative (A,C) and positive (B,D) controls**.

**Figure 6:**
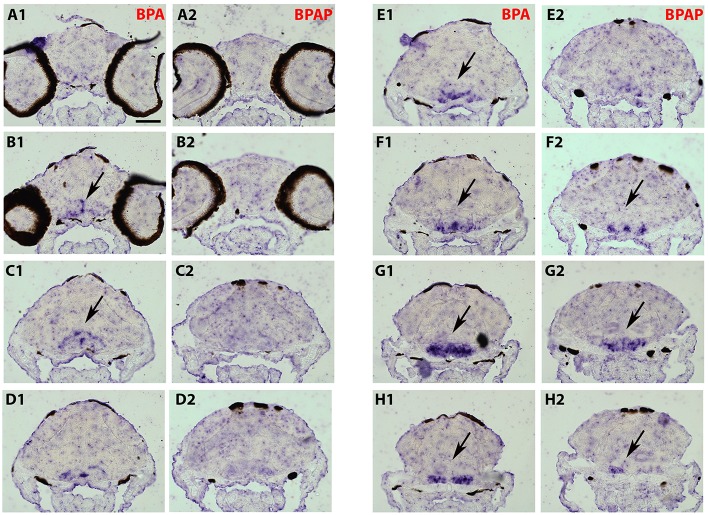
**Distribution of ***cyp19a1b*** transcripts in a 7-dpf old zebrafish brain after treatment with BPAP (A2–H2) and compared to a BPA-treated brain (A1–H1)**. Images of transverse sections through the rostrocaudal axis of brains. Arrowheads highlight areas of labeling. For all images, dorsal is to the top. Scale bar = 50 μm.

As the strongest induction of *cyp19a1b* transcripts along the rostro-caudal axis of the brain was observed in the caudal hypothalamus, notably in the area of the nucleus recessus posterioris (nrp) (Figures 4I2,3), we therefore focused our analysis on this brain particular region for the assessment of estrogenic activities of BPA analogs. As shown in the Figure 5, we confirmed that no *cyp19a1b* transcript was detectable in the nrp of DMSO-treated larva (Figures 5A,C), whereas strong levels of *cyp19a1b* transcripts were found in BPA-treated larva (Figures 5B,D). Most importantly, a similar high detection of c*yp19a1b* transcripts was also observed in this hypothalamic region with other bisphenols such as BPS, BPAF, and BPF (Figures 5E–H). These *in situ* data are in perfect agreement with the above RT-qPCR and EASZY assays (Figures 1, 2). For BPAP-treated larva, there was also an increase in *cyp19a1b* transcripts in the nrp region (Figure 5H) albeit to a lower levels compared to specimens treated with other bisphenols (BPA, BPS, BPF, and BPAF). Although no significant increase of *cyp19a1b* transcripts was observed in BPAP-treated animals using RT-qPCR and EASZY assays (Figures 1, 2), we decided to analyze in close details the distribution of *cyp19a1b* transcripts in the whole brain of BPAP-treated larva. As clearly shown in Figure 6, BPAP induces expression of *cyp19a1b* transcripts in only a few (xeno) estrogen-sensitive c*yp19a1b* expression sites. In particular, *cyp19a1b* transcripts were not observed in the anterior regions of the brain, corresponding to the posterior telencephalon and the preoptic area (Figures 6B2–E2). For BPS-, BPF-, and BPAF-treated animals, the *cyp19a1b* transcripts distribution patterns in the whole brain were identical to EE2- and BPA-treated animals, including in brain regions other than the nrp (dat not shown). Taken together, the absence of *cyp19a1b* transcripts in anterior regions of the brain and the weak detection of *cyp19a1b* transcripts in the caudal hypothalamus argue and confirm that BPAP has almost no estrogenic activity in the brain compared to other tested bisphenols (BPA, BPF, BPAF, and BPS).

### Bisphenols induce cyp19a1b activity through estrogen receptors in a glial cell model

**Figure 7:**
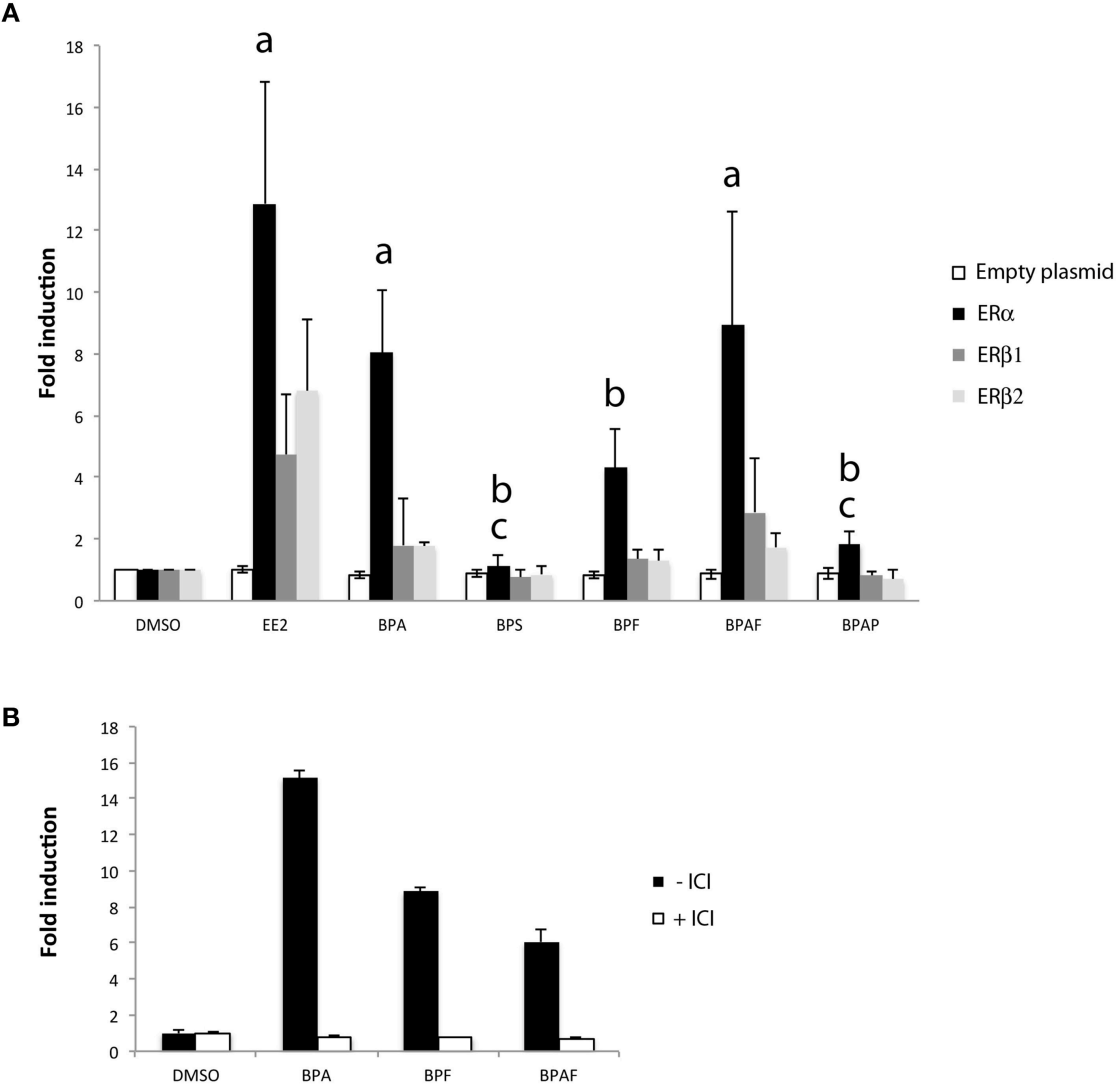
**(A)** *Cyp19a1b*-luciferase assay in a glial cell context expressing either ERα, ERβ1, or ERβ2. Luciferase activity was measured in triplicate for each condition following treatment with EE2 or bisphenol analogs. Data are presented as mean ± SEM of five independent cellular treatment experiments. **a**: *p* < 0.05 vs. DMSO in ERα–containing cells; **b**: *p* < 0.05 vs. EE2 in ERα-containing cells; **c**: ERα vs. BPA. **(B)** *Cyp19a1b*-luciferase assay in a glial cell context expressing the ERα sub-type in presence or absence of ICI. Luciferase activity was measured in triplicate for each condition.

To investigate in more detail the mechanism of bisphenols-induced transcription of *cyp19a1b* in radial glial cells of the brain, we performed functional *cyp19a1b*-luciferase reporter gene assay in a reconstituted glial cell line model (Le Page et al., 2006). We tested the impact of each bisphenol on the transcriptional activity of the three distinct zebrafish estrogen nuclear receptors (ERα, ERβ1, and ERβ2) transfected in U251MG cells, an ER-negative human glial cell line. In this assay, the zebrafish *cyp19a1b* promoter upstream of the luciferase is used as the reporter gene. We evaluated *trans*-activation properties of the different zebrafish estrogen nuclear receptors (ERα, ERβ1, and ERβ2) upon individual bisphenol treatments (BPA, BPS, BPF, and BPAF), EE2 and DMSO in six independent transfection experiments. As shown in Figure 7A, a priori analysis using a one-tailed *t*-test confirmed previous studies (Le Page et al., 2010) showing a strong induction of the *cyp19a1b*-luciferase reporter gene upon EE2 treatments in cells transfected with either ERα (*t* = 3.01 *df* = 10, *p* < 0.01), ERβ1 (*t* = 1.927 *df* = 10, *p* < 0.05) or ERβ2 (*t* = 2.495, *df* = 10, *p* < 0.05) compared to DMSO treatment. EE2-dependent activation of the *cyp19a1b* promoter was about 2- to 3-fold more efficient with ERα compared to ERβ1 and ERβ2. No reporter gene activity was found in absence of estrogen receptors (Figure 7A; empty plasmid), confirming that the transcriptional activity detected upon EE2 treatment is mediated by the presence of an estrogen nuclear receptor. We found a significant effect of the treatment [*F*_(6, 82)_ = 12.69, *p* < 0.0001], estrogen receptor subtype [*F*_(3, 82)_ = 24.24, *p* < 0.0001], and an interaction [*F*_(18, 82)_ = 3.53, *p* < 0.0001] on luciferase expression. More precisely, *post-hoc* analysis revealed a significant stimulation of *cyp19a1b* promoter activity by BPA and BPAF in ERα-containing cells. BPF also increased luciferase activity in ERα cells, but the stimulation did not reach statistical significance. In contrast, no significant luciferase activity was found in cell transfected with subtype receptor ERβ1 or ERβ2 upon stimulation with any bisphenols. Independently of the estrogen receptor sub-type, BPAP, and BPS did not stimulate the reporter gene (Figure 7A). To further confirm that stimulation of *cyp19a1b* promoter upon BPA, BPF, and BPAF treatments was mediated by ERα–dependent transcription, we repeated the transfection experiments with ERα in presence or absence of ICI 182 780, an antagonist of estrogen nuclear receptors. As shown in Figure 7B, simultaneous treatment with ICI completely abolished BPA, BPF, and BPAF stimulations found in ERα-expressing cells (Figure 7A). Taken together, the reporter gene assays in a glial cell context provide evidence that BPA, BPF, and BPAF are ERα agonists whereas BPS is likely to work through other mechanisms.

### Bisphenols binding to zebrafish estrogen nuclear receptors

**Figure 8:**
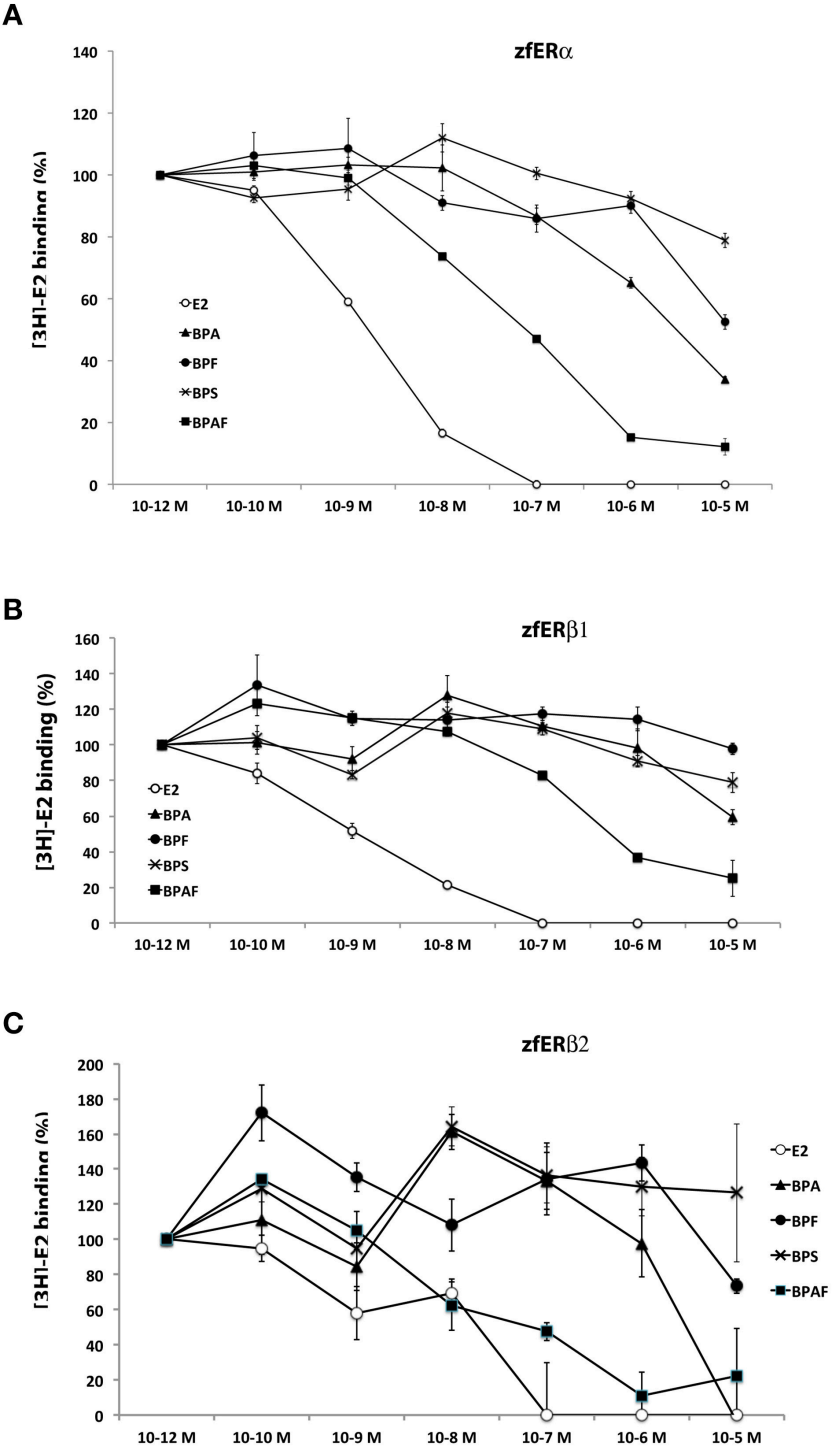
*****In** vitro* bisphenols-and E2-binding activity to zebrafish estrogen receptors**. **(A–C)** Competitive binding assays relative to [3H]17β-estradiol on ERα **(A)**, ERβ1 **(B)**, and ERβ2 **(C)**. Binding activity was measured in triplicate for each condition and data are presented as mean ± SEM of these 3 radioactivity countings. IC50 are provided in the Results section.

Using *in vitro* competition assays strategy, we examined the receptor-binding affinity of BPA and BPA analogs (BPS, BPA, BPF, and BPAF) relative to [3H]17beta-estradiol for the three *in vitro* translated zebrafish estrogen receptors (ERα, ERβ1, and ERβ2). As expected, E2 show a high binding activity with the three zebrafish estrogen receptors (IC50: 1.5, 1.1, and 1.2 nM for ERα, ERβ1, and ERβ2, respectively; Figures 8A–C). In perfect agreement with reporter gene assays, we found that BPA, BPF, and BPAF bind *in vitro* to ERα receptor (Figure 8A). BPAF showed the highest affinity, followed by BPA and BPF (IC50; 0.076, 2.8, 10.6 μM, respectively). In addition, these receptor-binding activities were clearly reduced with ERβ1 and ERβ2 sub-type receptors (Figures 8B–C). In the competition assay with BPAF, the IC50 was almost 10 times stronger for ERβ1 [IC50 = 0.66 μM] than for ERα. Importantly, BPS displayed almost no binding affinities with any ER receptors (Figures 8A–C). These results suggest that the weak *cyp19a1b*-luciferase activity observed in the transfection experiment for BPS was probably due to the absence of binding to ERα.

## Discussion

The present work investigated the effects of several bisphenol A analogs on *cyp19a1b* gene regulation, coding for aromatase B, a well-known target of (xeno) estrogen signaling pathways in the fish brain. We show here that BPA, BPS, BPF, and BPAF are able to up-regulate the aromatase B in the brain of developing zebrafish, using three different *in vivo* and *in situ* methods, i.e., RT-qPCR, *in situ* hybridization and the transgenic *cyp19a1b*-GFP.

The BPA exposure of zebrafish larvae led to a 36-fold over-expression of *cyp19a1b* gene in the brain compared to control 7-dpf larvae, as demonstrated by RT-qPCR (Figure 1B). This over-expression was confirmed by the EASZY assay with which we found a BPA-inducing activity of the *cyp19a1b* promoter by quantification of the GFP fluorescence in transgenic *cyp19a1b*-GFP brains in 4-dpf larvae (Figure 2B; 16-fold induction). The level of *cyp19a1b* induction is in agreement with previous RT-qPCR experiments carried out on whole zebrafish larvae that were exposed to BPA between 3 and 4-dpf developmental stages (Chung et al., 2011) and with previous experiments performed in our laboratory (Brion et al., 2012). Additionnally, *in situ* hybridization experiments on larva brain sections confirmed that BPA caused a strong expression of the *cyp19a1b* gene in specific areas of the brain, in particular in the hypothalamus (Figures 3–5). Thus, we show here that the combination of the three different techniques (RT-qPCR, EASZY assay, and *in situ* hybridization) is a valid strategy to investigate estrogenic properties of bisphenol compounds.

Our study indicates that BPA analogs, BPS, BPAF, and BPF, stimulate *cyp19a1b* expression *in vivo*. Indeed, in larvae treated with one of these three bisphenols, we observed a clear induction of the endogeneous *cyp19a1b* gene expression in the brain of wild type 7-dpf old zebrafish (Figures 1B, 5) and up-stimulation of the *cyp19a1b* promoter in radial glial cells of GFP transgenic zebrafish larvae (4 dpf; Figure 2). The pattern of up-regulation of *cyp19a1b* by the three BPA analogs is similar to the modulation observed after BPA exposure, and therefore, strongly suggest that BPS, BPAF, and BPF exert estrogenic effects on developing zebrafish brain. It is also likely that numerous other estrogen sensitive responses, in the brain but also in other tissues, will also be directly affected by the presence of the bisphenol analogs. Moreover, the up-regulation of brain aromatase will lead to an elevated local enzymatic activity and therefore, elevated levels of locally produced endogenous estrogen might be expected, adding to the direct estrogenic effect of bisphenol. This abnormal increase in estrogenic activity, considered as endocrine disrupting activity, will likely affect brain development at molecular, cellular, organ, and functional levels, as previously shown for BPA (Kajta and Wojtowicz, 2013; Rochester, 2013; Leon-Olea et al., 2014; Negri-Cesi, 2015). *In utero* or perinatal exposures to BPA in mammals leads to permanent disruptions in behavior, including increased levels of aggression and anxiety, and alterations in learning, memory, exploration, and emotional responsiveness (see for example, refereces Farabollini et al., 2002; Miyatake et al., 2006; Rubin et al., 2006; Kawai et al., 2007; Palanza et al., 2008; Tian et al., 2010; Galea and Barha, 2011; Wolstenholme et al., 2011; Xu et al., 2011). There is little information on potential *in vivo* effect of BPA analogs on brain development and function. However, studies at peripheric level provide evidence that BPA analogs can have adverse effects by interfering with the endocrine system. For instance, the balance of sex steroid hormones and normal reproduction was significantly affected in adult zebrafish following early (Naderi et al., 2014) or late (adult stage) exposure to BPS (Ji et al., 2013). In the later study, BPS exposure led to a significant increase of 17β-estradiol and decrease of testosterone in the plasma of male zebrafish, and these alterations were accompanied by an up-regulation of central and peripheral aromatase expression (both *cyp19a1a* and *cyp19a1b* genes). Similarly to BPS, BPAF exposure can also disrupt sex hormone levels and vitellogenin expression in zebrafish (Yang et al., 2014) and, in adult male rats, BPAF was associated with testosterone reduction by directly affecting testis function (Feng et al., 2012). In human fetal testis explants, low dose of BPS, or BPF is also sufficient to decrease basal testosterone secretion (Eladak et al., 2015). In the brain, it was recently shown that low-dose exposure to BPA and BPS might cause hyperactivity and brain changes in the developing zebrafish due to precocious hypothalamic neurogenesis (Kinch et al., 2015). Interestingly, such brain effects were paralleled with an increase of c*yp19a1b* expression. In *C. elegans*, BPA, and BPS exposure during early embryogenesis also affect neural functionality at adult stage (Mersha et al., 2015) and, in juvenile female rats, BPA, BPF, and BPS can affect 5α-reductase expression and dopamine-serotonin innervations in the prefrontal cortex (Castro et al., 2015).

The preoptic area and the hypothalamus are key integrative centers in the brain that play pivotal functions in the neuroendocrine regulation of homeostasis, reproduction, sexual behavior and stress response (Zohar et al., 2010 and references therein). The detailed analysis of the distribution of *cyp19a1b* transcripts on thin serial sections corresponding to the whole brain (7 dpf) provided evidence that exposure to xenoestrogenes, such as BPA or EE2, strongly induced expression of *cyp19a1b* transcripts in specific brain regions, including posterior telencephalon, preoptic area and caudal hypothalamus (Figures 3, 4). Increase in *cyp19a1b* promoter activity in these specific brain regions was also detected in transgenic larvae (EASZY assays; Figure 2). The different brain regions where *cyp19a1b* was upregulated following bisphenol exposure were previously identified as sites of estrogen-induced *cyp19a1b* expression (Menuet et al., 2005; Lassiter and Linney, 2007; Tong et al., 2009; Mouriec et al., 2009a; Chung et al., 2011). In addition, we demonstrated that the caudal hypothalamus, and more precisely the lateral and posterior ventricular recesses (nrp), contains the highest amount of *cyp19a1b* transcripts after BPA or EE2 exposures. Importantly, BPS, BPF and BPAF were also able to strongly induce *cyp19a1b* expression in the nrp in the caudal hypothalamus, with a similar intensity compared to BPA and EE2 (Figure 5). Since various xenoestrogens can strongly stimulate *cyp19a1b* expression in similar and specific brain regions, this raise concern about the consequences of their combined actions on hypothalamic development and functioning.

Three distinct nuclear estrogen receptors (zfER) are characterized in zebrafish: ERα, ERβ1, and ERβ2, corresponding to *esr1, esr2b*, and *esr2a*, respectively. The three receptors can bind estradiol and are strongly expressed in the anterior and posterior preoptic area, and in the caudal hypothalamus (Menuet et al., 2002). As shown in this study, these two neuroendocrine regions are also the major sites of bisphenol-induced *cyp19a1b* expression (Figures 3–6). To define which estrogen receptors was implicated in the up-regulation of *cyp19a1b* gene, we used zebrafish *cyp19a1b* promoter luciferase reporter gene assay in a reconstituted glial cell context expressing one zebrafish ER subtype at a time (as previously reported (Menuet et al., 2005; Le Page et al., 2006). We showed here that BPA, used at a 10^−6^ M concentration, activates *cyp19a1b* promoter via its interaction with ER alpha, while the presence of either ER beta subtypes does not allow BPA to activate the promoter. BPF and BPAF are also able to stimulate *in vitro* the *cyp19a1b* promoter activity via the activation of ERα. This up-regulation was inhibited by the presence of the specific estrogen receptor antagonist ICI 182 780. (Figure 7). *In vitro* ligand competition assays confirmed that BPF and BPAF, in addition to BPA, physically bind zebrafish estrogen receptor alpha (Figure 8). Previous *in vitro* studies (reporter gene and ligand binding assays) performed in other cellular models also showed the ability of BPF and BPAF to act as an estrogen mimic that binds to estrogen receptors and subsequently exert *trans*-activation activities (Kitamura et al., 2005; Cabaton et al., 2009; Matsushima et al., 2010; Li et al., 2012). In yeast assays, estrogenic activity for these BPA analogs was also reported (Hashimoto et al., 2001; Ruan et al., 2014).

As stated above, we showed that BPS significantly stimulated the expression of *cyp19a1b in vivo* but, interestingly, this effect did not involve the bisphenol-dependent activation of estrogen receptors in our *cyp19a1b*-luciferase reporter gene in the functional glial cell assay (Figure 7). This is in stark contrast with the data obtained with BPA, BPF, and BPAF, acting on *cyp19a1b* up-regulation via ERα subtype (Figure 7). It is possible that BPS has only a very weak affinity for the zebrafish estrogen receptors. This hypothesis was clearly reinforced by the ligand competition assays that showed almost no binding activity for BPS compared to other tested bisphenols (Figure 8). Previous *in vitro* studies have shown for BPS, lower (Chen et al., 2002; Kitamura et al., 2005; Rosenmai et al., 2014), similar (Hashimoto et al., 2001; Kitamura et al., 2005; Kuruto-Niwa et al., 2005; Grignard et al., 2012; Kang et al., 2014), or higher (Molina-Molina et al., 2013) estrogenic activity than BPA, depending on the experimental model. In the reporter gene assay, the lack of estrogenic activity for BPS could be linked to the absence of key transcriptional co-factors required for efficient estrogen receptor-dependent *trans*-activation. Alternatively, *in vivo* BPS-induced estrogenic activity could be mediated through a different pathway than estrogen receptors. BPS acts as an estrogen mimic in certain conditions, but can also antagonize androgen receptor (Hashimoto et al., 2001; Kitamura et al., 2005; Kuruto-Niwa et al., 2005; Grignard et al., 2012). Recent *in vitro* studies provide evidence that BPA analogs have a clear effect on androgen receptor activity as well as on steroid hormone synthesis (Rosenmai et al., 2014), suggesting that these compounds may interfere with the endocrine system through several modes of action. In addition, in the case of BPAF and in human breast cells, estrogenic activity has been proven to be mediated through both genomic (ERα) and nongenomic pathways (Li et al., 2014).

We did not observe a significant induction of the expression of *cyp19a1b* gene expression in the brain of larvae treated with BPAP, using RT-qPCR and EASZY strategies (Figures 1, 2). These data were confirmed in the glial cell context *cyp19a1b*-luciferase reporter gene assay (Figure 7). The detailed analysis of *cyp19a1b* transcripts distribution carried out in the whole brains of BPAP-treated larvae revealed that *cyp19a1b* transcripts were, indeed, not detected in telencephalon or preoptic area and very weakly in the caudal hypothalamus (Figure 6). Taken together, these data suggest that BPAP might have no or a very weak estrogenic activity in the brain *in vivo*, compared to other bisphenols tested here (BPA, BPS, BPF, and BPAF). To the best of our knowledge, there is only one study that reports low estrogenic activities for BPAP in a recombinant gene yeast assay (Zhang et al., 2009). Our data suggest that BPAP might be a safer alternative to BPA and to other BPA analogs currently used. Presently, BPAP use in industry is not (yet) a common BPA substitute as its environmental concentrations, as measured in food or in indoor dust, is very low compared to other bisphenols (Liao et al., 2012b; Liao and Kannan, 2013).

In conclusion, this work shows that BPA, BPF, BPAF, and BPS exhibit estrogenic activity on the *cyp19a1b* gene (aromatase B), a brain specific gene, which is considered as one of the most, if not the most, E2-sensitive gene in fish (Brion et al., 2012; Lee et al., 2012; Petersen et al., 2013). Thus, BPA analogs, because of their widespread use and their potential to persist in the environment, may be equally as harmul as BPA to developing brains. In contrast, BPAP appears to have no estrogenic activity in the brain of zebrafish. To confirm that BPAP could be a safer alternative to BPA, studies investigating its effects on other tissues and signaling pathways will be required. A replacemant of BPA by any of these coumpounds should be considered with caution and further studies are clearly required to clarify the precise *in vivo* effects of BPA analogs and their mechanisms of actions.

## Author contributions

PC, designed the study, developed the methodology, conducted experiments and wrote the manuscript. JC, developed methodology and conducted experiments. CV, maintained embryo and larva development and helped with qPCR analysis. TC, helped with the statistics and editing the article. OK, EP, helped with the design of the study and editing the article.

### Conflict of interest statement

The authors declare that the research was conducted in the absence of any commercial or financial relationships that could be construed as a potential conflict of interest.
